# Supplementary material for: Dual Application: p-CuS/n-ZnS Nanocomposite Construction for High-Efficiency Colorimetric Determination and Photocatalytic Degradation of Tetracycline in Water
Source: Nanomaterials (Basel). 2022 Nov 22;12(23):4123. doi: 10.3390/nano12234123 (PMC9737547; doi:10.3390/nano12234123)
Supplement: Supplementary file 1 [file nanomaterials-12-04123-s001.zip › nanomaterials-2038167-supplementary.pdf]

# Supplementary Information

# Dual Application: p-CuS/n-ZnS Nanocomposite Construction for High-Efficiency Colorimetric Determination and Photocatalytic Degradation of Tetracycline in Water

Li Zhang [a, b], Linhong Ge [a, b], Lamei Deng [a, b], Xinman Tu [a, b] *

*a Key Laboratory of Jiangxi Province for Persistent Pollutants Control and Resources Recycle, Nanchang Hangkong University, Nanchang 330063, People's Republic of China.*

*b National-Local Joint Engineering Research Center of Heavy Metals Pollutants Control and Resource Utilization, Nanchang Hangkong University, Nanchang 330063, People's Republic of China.*

* Corresponding author

*E-mail address*: tuxinman@126.com

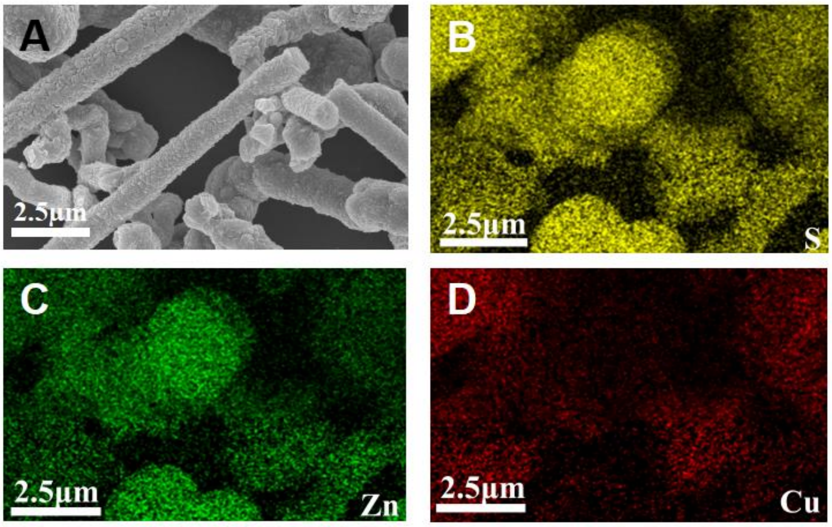


**Figure S1** (A) SEM images of ZnS and (B~D) elemental mappings of the prepared CuS/ZnS.

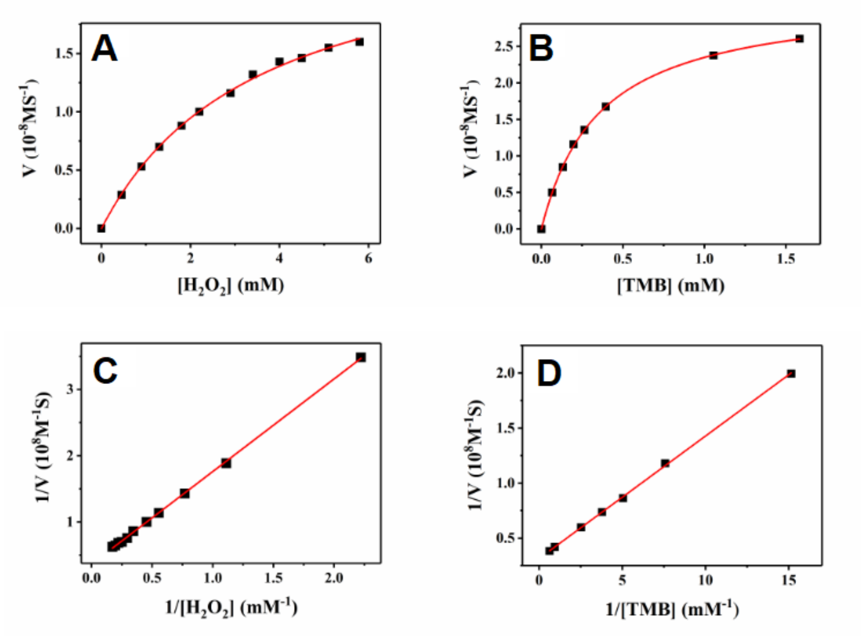


**Figure S2** (A and C) Steady-state kinetic assays of CuS/ZnS catalytic performance for different concentrations of $H_2O_2$ with 0.2 mM TMB, (B and D) that for different concentrations of TMB with 20 mM $H_2O_2$.

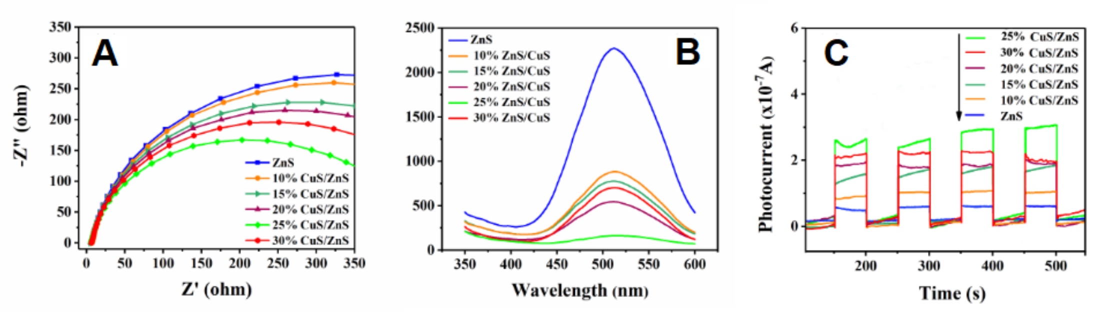


**Figure S3** Electrochemical impedance spectroscopy (A), photoluminescence spectra (B) and photocurrent spectra (C) of ZnS and CuS/ZnS composites with different mass ratio of CuS.

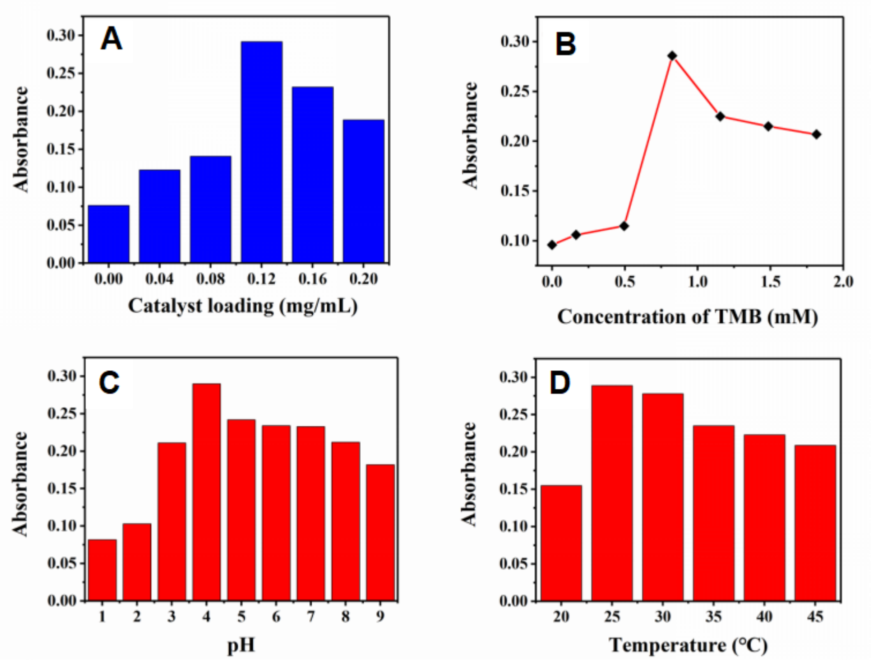


**Figure S4** The effect of catalyst loading amounts (A), TMB concentrations (B), pH values (C) and reaction temperatures (D) on the TC detection.

**Table S1** The comparison of the linear range and LOD of this strategy and other reported method for TC determination.

| Catalysts | Linear range（μM） | LOD（nM） | References |
|---|---|---|---|
| AuNCs-Apt | 1~16 | 46 | [1] |
| TH/NAD(P)H/TetX2 | 0.1~0.8 | 60 | [2] |
| AuNPs | 0.1~5 | 71 | [3] |
| $Fe_3O_4$ MNPs | 0.1~1 | 45 | [4] |
| CuS/ZnS | 0.25~3 | 20.94 | This work |

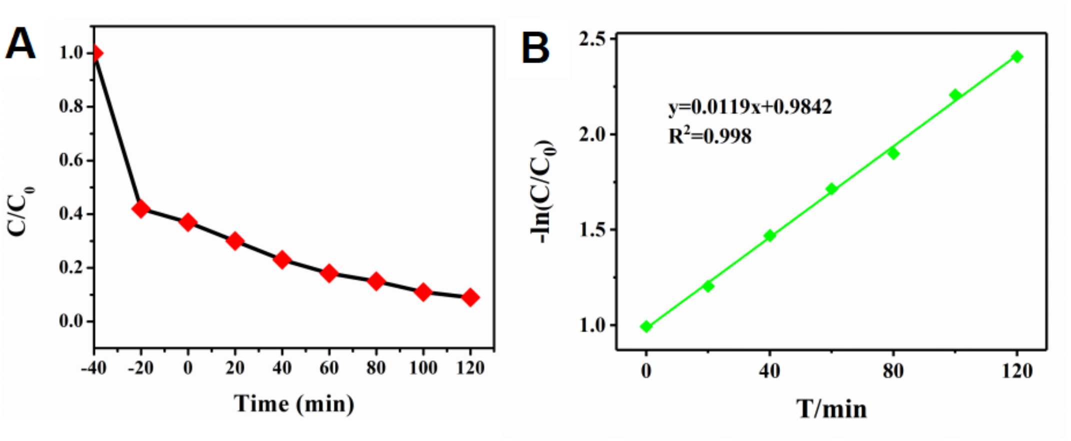


**Figure S5** (A) The degradation rate of the optimized CuS/ZnS to TC, (B) the apparent rate constant (K) plot of the degradation.

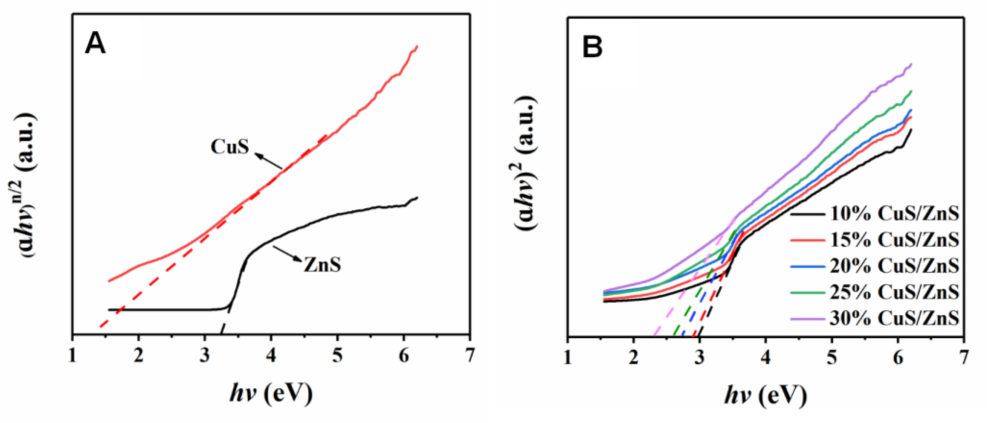


**Figure S6** (A) UV-Vis DRS graph of CuS and ZnS, (B) UV-Vis DRS graph of CuS/ZnS nanocomposite with different amount of CuS.

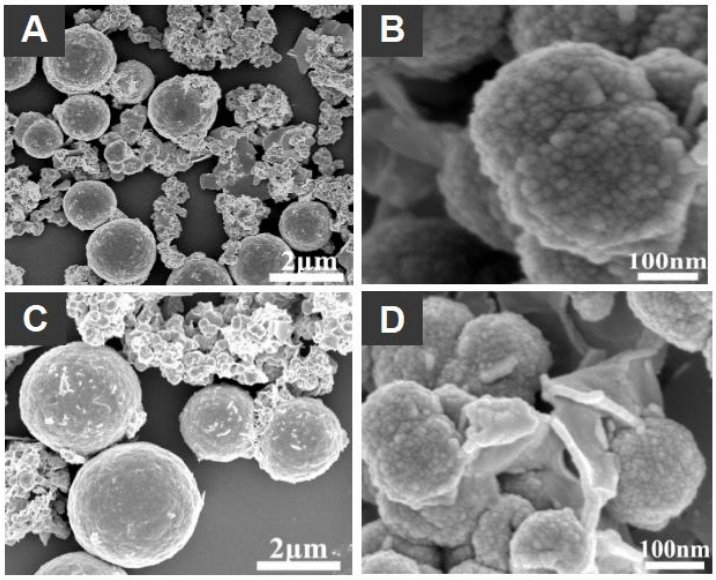


**Figure S7** The SEM images of the optimized CuS/ZnS before (A and B) and after (C and D) reuse.

## Reference


1. Zhang, Z.; Tian, Y.; Huang, P.; et al. Using target-specific aptamers to enhance the peroxidase-like activity of gold nanoclusters for colorimetric detection of tetracycline antibiotics. *Talanta*, **2020**, *208*, 120342.
2. Besharati, M.; Tabrizi, M.A.; Molaabasi F.; et al. Novel enzyme-based electrochemical and colorimetric biosensors for tetracycline monitoring in milk. Biotechnol. *Appl. Biochem.*, **2022**, *69*, 41-50.
3. Qi, M.; Tu, C.; Dai, Y.; et al. A simple colorimetric analytical assay using gold nanoparticles for specific detection of tetracycline in environmental water samples. *Anal. Methods*, **2018**, *10*, 3402-3407.
4. Wang, Y.; Sun, Y.; Dai, H.; et al. A colorimetric biosensor using Fe3O4 nanoparticles for highly sensitive and selective detection of tetracyclines. *Sens. Actuators B Chem.*, **2016**, *236*, 621-626.
